# Gut–Heart Axis and Infective Endocarditis: How Microbiota Dysbiosis Shapes Cardiovascular Risk and Infection Susceptibility

**DOI:** 10.3390/jcm15020597

**Published:** 2026-01-12

**Authors:** Livia Moffa, Claudio Tana, Tiziana Meschi, Carmine Siniscalchi, Nicoletta Cerundolo, Claudio Ucciferri, Jacopo Vecchiet, Katia Falasca

**Affiliations:** 1 Clinics of Infectious Diseases, University “G. d’Annunzio” of Chieti-Pescara, 66100 Chieti, Italy; 2 Internal Medicine Unit, Eastern Hospital, ASL Taranto, 74024 Manduria, Italy; 3 Internal Medicine Department, Parma University Hospital, 43126 Parma, Italy

**Keywords:** gut–heart axis, infective endocarditis, microbiota, dysbiosis, cardiovascular disease, microbiota-derived metabolites

## Abstract

The gut–heart axis represents a key determinant of cardiovascular (CV) system health. Emerging evidence indicates that intestinal dysbiosis can induce a state of chronic systemic inflammation which, together with mechanisms of endothelial dysfunction, increases the risk of CV diseases. Infective endocarditis (IE) exemplifies this concept, as microbiota alterations may promote bacterial translocation from the gut into the bloodstream, leading to colonization of cardiac valves and subsequent endocardial infection. This narrative review examines current scientific evidence on the relationship between the gut microbiota and CV diseases, with a particular focus on IE. We also summarize the mechanisms underlying impaired intestinal barrier integrity, immune activation, and the production of microbiota-derived metabolites that contribute to CV disease. Special attention is given to potential preventive and therapeutic strategies, including microbiota modulation, targeted antibiotic management, and personalized medicine approaches tailored to individual patient profiles.

## 1. Introduction

The gut–heart axis has emerged as a particularly compelling and rapidly evolving area within the pathophysiology of cardiovascular (CV) diseases [1,2]. The gut carries out multiple essential functions, including the regulation of immune responses, inflammatory homeostasis, and metabolic processes, largely through the activity of the intestinal microbiota [3]. The microbiota interacts continuously with the CV system, influencing key physiological pathways. Through the production of metabolites, modulation of inflammatory responses, and regulation of endothelial function, it contributes to both CV homeostasis and disease progression [4]. According to recent studies, several microbiota-derived metabolites such as trimethylamine-N-oxide (TMAO), short-chain fatty acids (SCFAs), and indole derivatives, play crucial roles in regulating inflammation, lipid metabolism, and endothelial function [5]. A balanced intestinal microbiota protects against chronic inflammation and the generation of mediators that drive atherogenesis. Gaining a deeper understanding of the gut microbiota, its metabolites, and their relationship with CV diseases may represent a turning point in both the prevention and treatment of these conditions [6,7]. An increasingly robust association between the gut microbiota and infective endocarditis (IE) is emerging. This condition arises from the interplay between endothelial injury and the translocation of pathogenic microorganisms into the bloodstream [8]. However, recent studies suggest that the composition of the intestinal microbiota itself may predispose certain individuals to an increased risk of IE. In particular, dysbiosis can impair the integrity of the gut barrier, facilitating bacterial translocation into the bloodstream [9]. A wide range of microorganisms can cause IE, and several of these originate from the gut, including *Streptococcus gallolyticus*, *Enterococcus faecalis*, and *Enterococcus faecium* [10].

Moreover, the intestinal microbiota may play a key role in modulating the immune system, influencing endothelial alterations and promoting the adhesion of bacteria to cardiac valves. This opens new pathophysiological perspectives linking gut health to systemic infections [11].

### Aims and Methods

The aim of this narrative review was to examine the complex interplay between the intestinal microbiota, CV risk, and susceptibility to IE by integrating the most up-to-date scientific evidence. In line with its narrative nature, this work seeks to synthesize emerging concepts and mechanistic insights rather than to provide a systematic or quantitative assessment of the literature. We also explored relevant clinical correlations and discussed emerging perspectives on microbiota-modulating strategies as potential avenues for prevention and management. A broad yet structured literature search was conducted across PubMed, Scopus, and Web of Science, using combinations of terms including ‘gut microbiota’, ‘dysbiosis’, ‘CV disease’, ‘atherosclerosis’, ‘infective endocarditis’, ‘bacterial translocation’, and ‘microbiota-derived metabolites’. Additional sources were identified through manual screening of reference lists and cross-referencing of key articles.

## 2. The Gut–Heart Axis

The intestinal microbiota performs several essential functions, including maintaining gut homeostasis through the production of specific metabolites that preserve the integrity of the intestinal barrier. Eubiosis is a balanced and healthy microbial ecosystem, and helps to limit inflammation and prevents microbial translocation. Dysbiosis, defined as an alteration in the normal gut microbial composition, has been linked to the development and progression of CV diseases [12]. In this context, dysbiosis can facilitate the passage of pathogen-associated molecular patterns (PAMPs) into the systemic circulation, triggering inflammatory responses and contributing to endothelial injury. Taken together, these mechanisms can form the biological foundation of the gut–heart axis [13]. Communication between the gut and the CV system appears to be bidirectional and can occur through multiple pathways. One key route can involve the vagus nerve, which conveys signals from enterocyte receptors to neural circuits that regulate vagal tone and heart rate. An endocrine pathway also can play a central role, as hormones produced by intestinal L-cells, such as glucagon-like peptide-1 (GLP-1) and peptide YY (PYY) can exert CV effects by modulating endothelial function, vascular tone, and lipid metabolism. In parallel, bile acid receptors including farnesoid X receptor (FXR) and Takeda G-protein–coupled receptor 5 (TGR5), can contribute to the regulation of lipid metabolism, inflammation, and vascular function, further supporting the bidirectional communication of the gut–heart axis [14]. Finally, an immune-mediated pathway also contributes to gut–heart communication. The intestinal microbiota can steer immune responses toward pro- or anti-inflammatory phenotypes. Several microbiota-derived metabolites, such as SCFAs, particularly acetate and butyrate, can modulate blood pressure through G-protein–coupled receptors (GPCRs) and the olfactory receptor Olfr78, promoting vasodilation and contributing to the regulation of vascular tone [15]. Indole-derived metabolites, such as indole-3-propionic acid, also exert cardioprotective effects in preclinical models through activation of the aryl hydrocarbon receptor (AhR), leading to reduced inflammation and improved diastolic function [16]. According to recent studies, an imbalance in bile acid composition and the underlying intestinal dysbiosis may increase the likelihood of atherosclerotic progression. Beyond atherosclerotic disease, emerging evidence from heart failure (HF) further supports the clinical relevance of the gut–heart axis. Patients with HF exhibit intestinal congestion, increased gut permeability, and profound alterations in gut microbiota composition, which favor microbial translocation and systemic inflammation. These changes have been linked to altered microbiota-derived metabolite signaling, immune activation, and disease progression, reinforcing the concept that gut dysbiosis may act as a modifier of CV vulnerability rather than a disease-specific trigger [17].

Although substantial evidence links gut microbiota alterations to CV disease, most findings are observational, and causal inference remains limited [18]. Beyond individual metabolites, emerging evidence highlights the existence of common, shared molecular pathways linking gut microbiota–driven metabolic dysfunction to CV risk. Alterations in host metabolic signaling, particularly involving insulin resistance, lipid handling, mitochondrial function, and low-grade systemic inflammation appear to represent convergent mechanisms through which dysbiosis may modulate CV disease susceptibility. These metabolically driven pathways may also indirectly influence endothelial vulnerability and infection risk, further reinforcing the systemic relevance of microbiota-related metabolic remodeling [19].

## 3. Intestinal Dysbiosis and CV Risk: Evidence from the Literature

One of the most extensively studied microbiota-derived metabolites is TMAO. In a landmark prospective study including 4007 patients undergoing elective coronary angiography, higher fasting plasma TMAO levels were independently associated with an increased 3-year risk of major adverse CV events (MACE: death, myocardial infarction, or stroke), with patients in the highest quartile showing a more than two-fold higher risk compared with those in the lowest quartile (hazard ratio [HR] ≈ 2.5) after adjustment for traditional risk factors [18].

Similar findings were observed in a cohort of 935 patients with peripheral artery disease (PAD), where higher fasting plasma levels of TMAO were strongly associated with increased 5-year all-cause mortality. Patients in the highest TMAO quartile had a 2.86-fold higher mortality risk compared with those in the lowest quartile, and this association remained significant after adjustment for traditional CV risk factors, inflammatory biomarkers, and history of coronary artery disease (CAD, adjusted HR 2.06). The prognostic value of elevated TMAO was consistent across different PAD subtypes, including carotid, non-carotid, and lower-extremity disease. Moreover, incorporating TMAO into clinical risk models significantly improved risk prediction, with a net reclassification improvement of 40.22% and an increase in the area under the ROC curve from 65.7% to 69.4% (*p* = 0.013). Overall, these findings indicate that TMAO is an independent predictor of long-term mortality in PAD and provides incremental prognostic information beyond established risk markers [20].

A systematic review which included 17 clinical studies enrolling 26,167 participants with a mean follow-up of 4.3 years, further confirmed a positive, graded association between circulating TMAO and both CV events and mortality. High TMAO concentrations were associated with a markedly increased risk of all-cause mortality (HR 1.91; 95% CI 1.40–2.61) and major adverse cardio- and cerebrovascular events (MACCE) (HR 1.67; 95% CI 1.33–2.11). A dose–response analysis further showed that each 10 μmol/L increase in circulating TMAO corresponded to a 7.6% rise in the relative risk of all-cause mortality (summary RR 1.07; 95% CI 1.04–1.11). Notably, the association between TMAO and mortality remained robust across all subgroups and populations examined. Collectively, these findings provide compelling evidence of a dose-dependent relationship between higher TMAO levels and increased CV risk and mortality [21].

Beyond TMAO, other microbiota-derived metabolites associated with dysbiosis also appear to modulate CV risk. A systematic review, which included 21 prospective studies encompassing 58,691 participants, found that branched-chain amino acids (BCAAs) demonstrated robust and consistent associations with increased CV risk, including higher incidence of CVD, myocardial infarction, and adverse events in patients with acute coronary syndromes. Findings for secondary bile acids were mixed, with some metabolites such as glycocholenate sulfate and glycocholate, linked to atrial fibrillation and mortality, while others showed no significant associations. Tryptophan was inversely associated with all-cause mortality, suggesting a potential protective role, and indoxyl sulfate was positively associated with MACE in patients with chronic kidney disease. No eligible prospective studies evaluated circulating SCFAs. Overall, the review supports a contributory role of specific microbiota-derived metabolites in CV risk, although the strength and direction of associations vary substantially across metabolite classes [22]. These data support the concept that dysbiosis-related metabolic remodeling can shift the host milieu toward a pro-atherogenic and pro-thrombotic state. Complementing metabolite-based evidence, several studies have examined the structure of the gut microbiota in patients with CAD. In a case–control study comparing 39 patients with CAD and 30 age- and sex-matched controls with similar risk factors, Emoto et al. used terminal restriction fragment length polymorphism (T-RFLP) and data-mining classification algorithms to examine gut microbiota structure. The analysis stratified microbial profiles into two major clusters and accurately distinguished CAD-specific patterns: 90% of CAD patients were assigned to CAD-associated microbiota nodes, while 93% of controls were assigned to control-specific nodes. Validation in an additional five CAD patients confirmed the model’s predictive accuracy. Three operational taxonomic units (OTU853, OTU657, and OTU990) emerged as key discriminative microbial signatures. Overall, the study demonstrates that gut microbiota composition differs markedly between CAD patients and controls and suggests that specific microbial patterns may serve as diagnostic markers for CAD [23].

More recently, large cohort studies have introduced the concept of ‘gut microbial age,’ showing that individuals whose microbiota appears metabolically healthier or ‘younger’ than expected for their chronological age exhibit a substantially lower long-term risk of CV events, even after accounting for conventional risk factors. In a discovery cohort of 10,207 adults aged 40–93 years, five metabolic multimorbidity clusters (MCs) were identified using 21 metabolic parameters, with the ‘obesity-related mixed’ cluster (MC4) and the ‘hyperglycemia’ cluster (MC5) showing markedly elevated 11.1-year CV risks of 75% (HR 1.75; 95% CI 1.43–2.14) and 117% (HR 2.17; 95% CI 1.72–2.74), respectively, compared with the metabolically healthy cluster (MC1). These associations were replicated in an independent cohort of 9061 individuals. Analysis of 4491 shotgun metagenomes demonstrated that gut microbial composition was strongly associated with both metabolic subphenotypes and aging, enabling the development of a validated gut microbial age (MA) metric based on 55 age-related microbial species. Among individuals ≥60 years, the excess CV risk linked to metabolically unhealthy phenotypes (MC4 and MC5) was amplified in those with a high MA but attenuated in those with a low, ‘younger’ MA, independent of age, sex, lifestyle, and diet. These findings suggest that a biologically younger gut microbiota can buffer the CV risk imposed by metabolic dysfunction, highlighting gut MA as a potential modulator of CV health in older adults [24].

## 4. Gut Dysbiosis and Infective Endocarditis: Mechanistic Links

The pathophysiology of IE is influenced by multiple factors, including intestinal dysbiosis. Alterations of the gut microbiota can promote the production of pro-inflammatory metabolites, leading to impairment of the intestinal barrier. This barrier is composed of tight junctions, the mucin-rich mucus layer, and the immune-surveillance system. Disruption of any of these components may facilitate the translocation of lipopolysaccharides (LPS) into the systemic circulation [25].

Recent studies have shown that intestinal dysbiosis is directly associated with an increased risk of bacteremia, suggesting that gut microbiota composition can influence susceptibility to systemic infections. In one study involving 42 patients with bloodstream infections (BSIs) and 19 healthy controls, analyzed through 16S rRNA gene sequencing, bacterial diversity was significantly reduced in BSI patients compared with controls (*p* < 0.001), and beta diversity demonstrated a clear separation between the two groups (PERMANOVA, *p* = 0.001). Four keystone genera, *Roseburia*, *Faecalibacterium*, *Prevotella*, and *Enterococcus* (LDA > 4), differed significantly, and, notably, the reduction of SCFA-producing bacteria such as *Roseburia* and *Faecalibacterium*, together with the overgrowth of potentially pathogenic taxa, appeared to compromise gut barrier integrity and increase host vulnerability, predisposing individuals to BSIs [26].

In the context of IE, gut dysbiosis may facilitate the overgrowth of specific bacterial taxa such as *Streptococcus gallolyticus*, *Enterococcus faecalis*, and *E. faecium,* that are capable of translocating into the bloodstream. Beyond this biologically plausible and largely observational framework, a recent bidirectional Mendelian randomization study has attempted to explore potential causal links between gut microbiota composition and IE. The analysis suggested that *Blautia* and *Ruminococcus2* were associated with an increased genetic susceptibility to IE. Notably, the bidirectional design also indicated that IE itself may negatively affect specific gut microbial taxa, supporting the concept of a reciprocal host–microbiome interaction. However, although Mendelian randomization strengthens causal inference compared with conventional observational studies, these findings should still be interpreted with caution and regarded as partly hypothesis-generating, given the complexity and dynamic nature of host–microbiome interactions [8]. Once in the circulation, these microorganisms can adhere to cardiac valves, particularly in the presence of contributing factors such as microlesions, degenerative changes, or prosthetic material. The development of IE requires indeed a permissive cardiac substrate, which includes endothelial injury, degenerative or calcific valvular disease, and the presence of prosthetic material or intracardiac devices [27]. Systemic inflammatory signaling associated with gut dysbiosis, mediated by microbial translocation, circulating pathogen-associated molecular patterns, and cytokine activation may promote endothelial activation and the formation of a prothrombotic or microthrombotic surface, thereby facilitating bacterial adhesion during episodes of bacteremia. In this framework, dysbiosis-related immune activation might be considered not as an independent cause of IE, but as a modifier of host susceptibility that could act in concert with established valvular pathology and foreign material to create a biologically permissive environment for infection [27].

Additionally, dysbiosis might reduce the host’s ability to clear bacteria from the bloodstream and creates a pro-inflammatory milieu characterized by enhanced neutrophil activity, Th17-skewed responses, elevated IL-6 levels, and macrophage priming, amplifying susceptibility to infection. These alterations are even more pronounced in older and frail individuals due to pro-inflammaging, a chronic low-grade inflammatory state that impairs both innate and adaptive immunity. The convergence of reduced bacterial clearance, dysregulated immune signaling, bacterial translocation, and age-related immune dysfunction may therefore play a pivotal role in the development of IE, especially in patients with predisposing cardiac conditions [28].

## 5. Microbial Metabolites in Infective Endocarditis: Beyond Atherosclerosis

In recent years, increasing evidence from the literature has highlighted the significant role of the gut microbiota and its metabolites in CV pathophysiology. These metabolites, including SCFAs, TMAO, and indole-3-propionate (IPA), are known to modulate immune responses and endothelial function. While these mechanisms are supported by robust experimental and CV evidence, their direct relevance to IE remains speculative and should currently be regarded as hypothesis-generating. TMAO is produced by the gut microbiota from dietary choline and L-carnitine, and elevated circulating levels have been associated with endothelial dysfunction in several experimental studies [29]. This metabolite promotes the generation of reactive oxygen species and activates pro-inflammatory signaling pathways. Preclinical studies have also demonstrated that TMAO exacerbates valvular calcification and aortic remodeling, promoting valvular fibrosis through mechanisms related to endoplasmic reticulum stress [30].

Although direct evidence linking TMAO to the risk of IE is currently lacking, endothelial dysfunction is widely recognized as a permissive substrate for bacterial adhesion to cardiac valves in the presence of bacteremia, consistent with recent reviews addressing the interplay between gut microbiota, valvular disease, and infection risk [31].

SCFAs, such as acetate, butyrate, and propionate, represent another key group of microbial metabolites involved in maintaining intestinal barrier integrity and regulating immune homeostasis. Butyrate, in particular, supports intestinal tight junctions and exerts protective effects on vascular function [32]. SCFAs have also been shown to inhibit biofilm formation by oral streptococci. However, their effects are concentration-dependent, and under certain pathological conditions, SCFAs may contribute to unfavorable mechanisms. Thus, SCFAs act as dynamic immunomodulators, whose protective or detrimental impact on valvular risk depends on the balance between eubiosis and dysbiosis [33].

IPA is a gut microbiota–derived metabolite that has demonstrated CV protective effects in recent studies. Beyond its vascular actions, IPA contributes to the maintenance of intestinal barrier integrity and, in animal models, has been shown to enhance phagocytic activity and provide protection against sepsis [34]. Although direct evidence linking IPA to IE is currently lacking, this metabolite appears to play a central role in regulating intestinal permeability, antimicrobial defenses, and systemic inflammatory responses [16]. Collectively, these mechanisms are key components in the pathophysiological pathway linking gut dysbiosis to bacteremia and, potentially, to valvular colonization.

Bile acids constitute an additional molecular link between the gut microbiota and CV disease. The intestinal microbiota converts primary bile acids into secondary bile acids, which act as ligands for receptors expressed on immune and endothelial cells [35]. Activation of these pathways modulates vascular function and stress responses, exerting protective effects in several CV conditions, including atherosclerosis. Alterations in bile acid metabolism may impair receptor signaling and increase intestinal permeability [16,35]. Moreover, several bacterial species commonly implicated in IE, such as enterococci and streptococci, exhibit high bile acid tolerance, facilitating their gastrointestinal colonization [36]. Recent studies on *Streptococcus gallolyticus* suggest that interactions with bile acids may enhance bacterial growth and translocation; however, the specific role of bile acids in IE remains poorly explored [37].

Overall, these microbiota-derived metabolites can influence intestinal permeability and inflammatory responses. Although direct evidence in IE remains limited, the bidirectional immunomodulatory effects of short-chain fatty acids suggest a plausible mechanistic link through which the gut microbiota may influence host conditions associated with either increased susceptibility to, or protection against, the development of IE.

## 6. Clinical Correlates: Which Patients Are Most Vulnerable?

Certain age groups are particularly predisposed to the development of infectious diseases affecting the CV system, with older adults representing a high-risk population. With advancing age, the gut microbiota undergoes profound alterations, including a reduction in microbial diversity, impairment of intestinal barrier integrity, and the development of a chronic low-grade inflammatory state. This phenomenon, commonly referred to as inflammaging, reflects the progressive dysregulation of immune and inflammatory pathways associated with aging and may contribute to increased susceptibility to CV infections [38]. Clinically, this vulnerability may be particularly relevant in older patients presenting with recurrent or unexplained bacteremia, especially in the presence of degenerative valvular disease or intracardiac devices, where transient bloodstream infections may more easily translate into endocardial colonization.

Conditions such as diabetes and obesity are also associated with gut microbiota alterations that favor a pro-inflammatory phenotype and increased susceptibility to infections. In obesity and type 2 diabetes, intestinal dysbiosis is characterized by changes in microbial composition, reduced diversity, and increased intestinal permeability, which promote chronic low-grade inflammation and systemic endotoxemia due to translocation of microbial products such as LPS [39]. These alterations may contribute to immune dysregulation and impaired host defense mechanisms, potentially facilitating recurrent or subclinical bacteremia. In individuals with advanced valvular heart disease, prosthetic valves, or a prior history of IE, the coexistence of metabolic dysfunction and systemic inflammation may define a clinically recognizable high-risk phenotype, in which dysbiosis-driven immune activation acts as a susceptibility modifier rather than a direct cause of infection [27,39,40]. In the presence of pre-existing valvular disease or intracardiac devices, this pro-inflammatory milieu may create a biologically permissive environment for endothelial activation, bacterial adhesion, and colonization of the endocardial surface during episodes of bacteremia, thereby indirectly increasing susceptibility to CV infections, including IE [39,40]. In such contexts, microbiome-informed hypotheses may be most plausibly explored within prevention-oriented research frameworks, rather than immediate therapeutic application. An overview of the key mechanisms and research priorities linking gut dysbiosis to CV disease and IE is provided in Table 1.

## 7. Diagnostic and Predictive Tools Based on the Microbiota

At present, no validated studies have identified specific microbial patterns associated with the development of IE [40]. Nonetheless, the possibility of identifying gut microbial clusters linked to an increased propensity for bacterial translocation and endothelial infection represents an area of growing interest [8].

In this context, metagenomic approaches, particularly shotgun sequencing, offer a powerful tool to explore these hypotheses by enabling high-resolution profiling of the gut microbiome at both taxonomic and functional levels [41]. Unlike targeted sequencing methods, metagenomics allows the identification of microbial genes involved in virulence, adhesion, biofilm formation, antimicrobial resistance, and metabolic pathways that may influence host immune responses and endothelial integrity [42]. By integrating metagenomic data with clinical and inflammatory phenotypes, it may become possible to identify microbial configurations associated with increased bacterial translocation and systemic exposure [43]. Furthermore, functional metagenomic analyses can provide insight into microbiota-derived metabolic and inflammatory pathways potentially involved in endothelial activation and susceptibility to infection. In high-risk populations, such as patients with valvular heart disease or prosthetic valves, these approaches could support the development of microbiome-based risk stratification models and inform targeted preventive strategies. Although this field remains largely exploratory, metagenomics represents a promising avenue for advancing our understanding of the gut–heart axis and its potential role in the pathogenesis of IE.

## 8. Therapeutic Modulation of the Microbiota

Interventions targeting the gut microbiota differ substantially in terms of clinical validation and translational readiness, ranging from established, evidence-based preventive strategies to experimental approaches that remain investigational, particularly in high-risk populations such as patients with IE.

Modulation of the gut microbiota as a strategy to manage CV diseases and IE represents one of the most complex yet compelling challenges in contemporary medicine; however, its application to IE remains largely theoretical and insufficiently supported by direct clinical evidence. At present, established approaches mainly include lifestyle interventions and the use of pharmacological agents capable of influencing the gut microbiota. Nevertheless, particular caution is required in specific populations, such as frail or immunocompromised patients, in whom such interventions should be carefully evaluated and judiciously applied [44].

Dietary intervention represents the most established strategy for gut microbiota modulation. A Mediterranean diet, rich in dietary fiber, is associated with increased microbial diversity and enhanced production of SCFAs. Conversely, reducing the intake of foods rich in L-carnitine may decrease the generation of TMAO [45]. These dietary measures may therefore contribute to a reduction in CV disease risk; however, with respect to IE, their potential impact remains largely hypothetical, as systematic studies specifically addressing this condition are currently lacking [45]. With regard to probiotics, prebiotics, and synbiotics, evidence suggests that these interventions may reduce bacterial translocation and systemic inflammation. However, there are currently no sufficient data to support their routine use in the management or prevention of IE [46].

Among strategies aimed at preserving or modulating the gut microbiota, antimicrobial stewardship represents the most robust and evidence-based intervention [2,28]. While antibiotics are indispensable for the treatment of infections, they are also a major driver of intestinal dysbiosis, with potential long-term consequences for immune homeostasis and endothelial vulnerability [28]. This balance is particularly critical in patients at increased risk of IE, such as those with prosthetic valves, intracardiac devices, or frequent healthcare exposure, in whom minimizing unnecessary or prolonged broad-spectrum antibiotic use may represent a key preventive principle to ensure effective therapy while preserving intestinal microbial homeostasis [28].

Although fecal microbiota transplantation has established indications in selected clinical settings, its role in patients with CV diseases or in those at increased risk of IE remains uncertain and insufficiently explored, precluding any clinical recommendation at present [47]. Emerging microbiota-targeted therapies, including inhibitors of microbiota-derived metabolites such as TMAO, have shown encouraging results in preclinical models; however, these findings remain experimental and cannot yet be translated into clinical strategies for IE, requiring validation through well-designed, disease-specific human studies [46,47].

Overall, therapeutic modulation of the gut microbiota represents a promising yet still evolving strategy. Current evidence supports the role of dietary interventions, whereas more advanced microbiota-targeted therapies require further investigation through well-designed controlled clinical trials. In the context of IE, these approaches should currently be regarded as adjunctive and supportive strategies rather than as direct therapeutic or preventive interventions, pending the availability of more robust clinical evidence.

## 9. Conclusions and Future Perspectives

The gut–heart axis represents a rapidly evolving field with significant implications for CV medicine and infectious diseases. While a growing body of evidence supports the involvement of gut microbiota–derived metabolic and inflammatory pathways in CV disorders, their specific relevance to IE remains largely unexplored and represents a major knowledge gap. To date, direct human data linking gut microbiota composition or microbiota-derived metabolites to IE risk are scarce, and most available evidence is indirect, associative, or hypothesis-generating. Although emerging genetically informed approaches, such as Mendelian randomization, provide causally informative signals, they do not yet allow definitive conclusions regarding the role of gut dysbiosis in IE pathogenesis.

Beyond mechanistic insights, this framework raises important clinical and translational questions. If gut dysbiosis contributes to endothelial vulnerability and systemic inflammation, future studies will need to determine whether gut health assessment should become part of comprehensive risk stratification in patients with structural heart disease or prosthetic valves, and how such evaluation could be implemented in clinical practice. Similarly, the concept of gut MA suggests the possibility that microbiome-targeted strategies aimed at restoring a more favorable metabolic profile could eventually emerge as preventive approaches in selected high-risk populations, particularly older adults.

Advancing this field will require a shift beyond descriptive metagenomic profiling toward integrative multi-omics approaches, combining metabolomics, proteomics, microbial functional analyses, and host-derived data, together with artificial intelligence (AI) and machine learning techniques capable of integrating complex biological and clinical datasets. In parallel, the implementation of long-term longitudinal studies will be essential to clarify temporal relationships and to determine whether specific microbial configurations precede disease onset or arise as downstream consequences of cardiovascular or infectious pathology. In this context, AI-based models may support risk stratification and personalized prevention strategies; however, an additional challenge will be to balance predictive performance with mechanistic interpretability, ensuring that computational outputs meaningfully inform biological understanding and clinical decision-making.

Accordingly, the gut–heart–IE axis should be interpreted as a unifying, hypothesis-generating framework. While biologically plausible and supported by indirect human and mechanistic evidence, definitive causal validation and disease-specific therapeutic implications remain important priorities for future multidisciplinary and translational research.

## Figures and Tables

**Table 1:** Key mechanisms, clinical correlates, and translational perspectives linking gut microbiota dysbiosis to cardiovascular disease and IE along the gut–heart axis. The table summarizes biological pathways and current knowledge gaps, highlighting diagnostic and therapeutic opportunities as well as future research priorities. Abbreviations: BA, bile acids; CV, cardiovascular; CVD, cardiovascular disease; FXR, farnesoid X receptor; IE, infective endocarditis; IPA, indole-3-propionic acid; LPS, lipopolysaccharide; ML, machine learning; PAMPs, pathogen-associated molecular patterns; SCFAs, short-chain fatty acids; TGR5, Takeda G-protein–coupled receptor 5; TMAO, trimethylamine-N-oxide.

Theme	Biological Rationale	Evidence Base	Clinical Relevance	Unmet Needs & Research Priorities
Dysbiosis and loss of barrier integrity	Reduced diversity, leaky gut, PAMP/LPS spillover	Consistent links with systemic inflammation and CV phenotypes	May favor bacteremia and endothelial vulnerability	IE-focused prospective cohorts are missing
Microbiota-derived metabolites shaping vascular risk	TMAO (pro-inflammatory); SCFAs/IPA (immune and barrier modulation)	Strong CV evidence for TMAO; supportive data for SCFAs/IPA	Indirect contribution to IE susceptibility	No validated metabolite signatures in IE
BA signaling at the immune–endothelial interface	FXR/TGR5 pathways; bile tolerance of gut pathogens	Increasing evidence in atherosclerosis and CVD	Possible role in bacterial colonization/translocation	Specific BA–IE mechanisms largely unexplored
Host immune tone and inflammaging	Th17 skewing, neutrophil priming, impaired clearance	Robust evidence in aging and metabolic disease	Higher infection susceptibility in frail patients	Need immune–microbiome studies in IE
High-risk clinical phenotypes	Valvulopathies, prosthetic valves, diabetes, obesity	Epidemiological and mechanistic plausibility	Targets patients for preventive strategies	Microbiome-based stratification lacking
Microbiome-enabled diagnostics	Shotgun metagenomics for taxonomic/functional profiling	Validated in cardiometabolic research	Identification of pro-translocation profiles	No IE-specific microbial signatures
Multi-omics functional phenotyping	Integration of metagenomics, metabolomics, proteomics	Emerging translational evidence	Links microbiota functions to clinical phenotypes	Standardization and validation needed
AI-driven predictive modeling	Machine learning integration of omics and clinical data	Proof-of-concept studies	Risk prediction for CV events and IE	Requires large longitudinal datasets
Therapeutic microbiota modulation	Diet, AMS, probiotics, emerging inhibitors	Diet supported; others heterogeneous	Supportive role in inflammation reduction	No evidence for routine IE prevention
Longitudinal causality	Temporal link between dysbiosis and disease onset	Currently limited data	Essential to infer causality	Priority for future research

## Data Availability

Not applicable, as no new data were generated.
